# Supplementary material for: Complexome profiling of the Chlamydomonas psb28 mutant reveals TEF5 as an early PSII assembly factor
Source: Plant Cell. 2025 Mar 17;37(6):koaf055. doi: 10.1093/plcell/koaf055 (PMC12164590; doi:10.1093/plcell/koaf055)
Supplement: koaf055_Supplementary_Data [file koaf055_supplementary_data.zip › psb28_interactive.zip!/plots/intensity/qualityPlots/peptides/CYN37_Cre06.g303300.t1.2.html]

### Cre06.g303300.t1.2

Trivial name: CYN37  
SeqCoverage [median]: 9.30  
Mass: 42.28 kDa  
  
 

| Sequence | MissedCleavage | Mass [Da] | wtIntensity | muIntensity | Score |
| --- | --- | --- | --- | --- | --- |
| NVKEGLYDGR | 1 | 1149.58 | 287.40 | 0.00 | 39.15 |
|  |  |  | 287.4 | 0.0 |  |
